# Editorial: Case Reports in general cardiovascular medicine: 2024

**DOI:** 10.3389/fcvm.2026.1962442

**Published:** 2026-08-24

**Authors:** Anna Cantore, Riccardo Accioli, Viola Salvini, Decoroso Verrengia, Maurizio Acampa, Junjie Xiao, Pietro Enea Lazzerini

**Affiliations:** 1 Department of Medical Sciences, Surgery and Neurosciences, University of Siena, Siena, Italy; 2 Division of Internal Medicine and Geriatrics, Azienda Ospedaliera Universitaria Senese, Siena, Italy; 3 Stroke Unit, Department of Emergency-Urgency and Transplants, Azienda Ospedaliera Universitaria Senese, Siena, Italy; 4 School of Medicine, Institute of Geriatrics (Shanghai University), Affiliated Nantong Hospital of Shanghai University (The Sixth People’s Hospital of Nantong), Shanghai University, Nantong, China

**Keywords:** aneurysm, cardiac rupture, cardiovascular diseases, coronary artery disease, hypertrophic cardiomyopathy, myocarditis, pericarditis

Cardiovascular medicine is a dynamic and evolving field including a wide range of conditions, treatments, and patient outcomes. Despite significant improvements in diagnosis and therapy in recent years, many challenges remain in the complex management of cardiovascular diseases. In this editorial, we provide an overview of the key findings emerging from the case reports published within the Frontiers in Cardiovascular Medicine Research Topic “*Case Reports in General Cardiovascular Medicine: 2024*.” This special issue collects 17 reports that provide valuable insights into atypical presentations, diagnostic challenges, and personalized therapeutic strategies across the complex spectrum of cardiovascular medicine (Table 1).

**Table 1:** Key findings from the articles published in the research topic.

Title	Authors	What is new?
Takotsubo syndrome/Coronary artery disease		
Takotsubo syndrome with apical thrombosis associated with hyperthyroidism crisis: a case report from high-altitude Tibet	Zhang et al.	Combined effects of severe thyrotoxicosis and chronic high-altitude hypoxia may induce Takotsubo syndrome by enhancing sympathetic activity and catecholamine responsiveness
Case Report: Onset of Takotsubo syndrome during a heart rehabilitation session	Jiménez-Jiménez et al.	Structured physical exercise may be an atypical but relevant trigger of stress-induced myocardial dysfunction
Case Report: Sudden cardiac death due to spasm of multiple coronary arteries	Tang et al.	A fulminant presentation of coronary artery spasm may result in refractory cardiac arrest and fatal cardiogenic shock
Case Report: The Parkes-Weber syndrome in the patient who underwent coronary surgery	Milosevic et al.	The presence of both Parkes–Weber syndrome could be a potential cause of cardiac dysfunction
Hypertrophic cardiomyopathy		
Case Report: Practice of whole-course pharmaceutical care: a case in hypertrophic cardiomyopathy and a review of the pharmacist's role	Li et al.	Genetic testing should be integrated into the diagnostic-therapeutic pathway of hypertrophic cardiomyopathy
Case Series: Genetic mimics of hypertrophic cardiomyopathy in elderly	Chumakova et al.	Age-related conditions may mimic the clinical presentation of hypertrophic cardiomyopathy, making phenocopies challenging to diagnose
Case Report: Dual-chamber pacemaker for hypertrophic cardiomyopathy with bradyarrhythmia and idiopathic pericardial effusion: a report of two cases and literature review	Liu et al.	The use of dual-chamber pacemakers offers a promising approach to managing hypertrophic cardiomiopathy,
Unusual hypertrophic cardiomyopathy: case report of an early onset wild-type ATTR amyloidosis accompanied by a chromosomal duplication involving the *MYH6* and *MYH7* gene	Hamidi et al.	The unprecedented coexistence of wild-type transthyretin amyloidosis and a rare MYH6/MYH7 chromosomal duplication, contributes to an unusually severe hypertrophic cardiomyopathy phenotype
Pericarditis		
Purulent pericarditis caused by *Nocardia*: a case report and literature review	Zhong et al.	Nocardia pericarditis could mimic tuberculosis pericarditis in immunocompromised patients
Case Report: Extrapulmonary tuberculosis presenting as multiple caseous pericardial masses	Wang et al.	Tuberculous pericarditis may present with multiple large caseous pericardial masses, a rare manifestation that can resemble malignancy
Myocarditis		
Eosinophilic granulomatosis with polyangiitis; a distinctive presentation with myocarditis and autoimmune haemolyticanaemia: case report	Ayish et al.	Eosinophilic granulomatosis with polyangiitis-associated myocarditis may significantly improve with early immunosuppressive therapy
Case report: A case of acute pancreatitis with myocarditis and thyrotoxicosis	Fu et al.	Acute pancreatitis can coexist with myocarditis and thyrotoxicosis, with complete clinical recovery following treatment
Cardiac rupture		
Case Report: Diverticulum of the left ventricular outflow tract: multimodal imaging	Chen et al.	A left ventricular diverticulum located on the lateral wall of the left ventricular outflow tract was identified after an initial misdiagnosis of aortopulmonary septal defect
Case Report: Rupture of the cardiac free wall after myocardial infarction confirmed by aortic computed tomography angiography	Liu et al.	Aortic computed tomography angiography may aid in the diagnosis of post-infarction cardiac free wall rupture, particularly in patients with severe clinical manifestations disproportionate to coronary findings
Case Report: Fatal atrioesophageal fistula following atrial fibrillation ablation—critical reflections on prevention	Dai et al.	Atrioesophageal fistula after atrial fibrillation ablation may cause both ischemic stroke and myocardial infarction
Aneurysm/pseudoaneurysm		
Complicated mycotic saccular aneurysm of the infra-renal abdominal aorta with infected retroperitoneal hematoma: a clinical case report	Chinaliyev et al.	An infected retroperitoneal hematoma complicating abdominal aortic aneurysm rupture represents a rare and challenging condition requiring multidisciplinary treatment
Case Report: Ascending hope—urgent endovascular repair of aortic pseudoaneurysm	Ronen et al.	Percutaneous endovascular repair can be a feasible alternative for high-risk patients with ascending aortic pseudoaneurysm when conventional surgery is contraindicated

Chest pain accounts for approximately 5% of all emergency department admission (1). Nevertheless, fewer than 10% of patients presenting with chest pain receive a diagnosis of acute coronary syndrome (2). Notably, up to 70% of patients undergoing coronary angiography because of angina and evidence of myocardial ischemia do not exhibit obstructive coronary artery disease (3). Indeed, acute coronary syndrome-like presentations may result from coronary functional disorders, such as epicardial or microvascular spasm, congenital coronary anomalies, or stress-related myocardial conditions such as Takotsubo syndrome (4). Prompt recognition is essential as delayed diagnosis or failure to initiate targeted treatment increases the risk of major adverse cardiovascular events (5). In this context, several compelling case reports shed light on unusual conditions that may mimic acute coronary syndrome. Zhang et al. described a rare case of Takotsubo syndrome complicated by left ventricular apical thrombosis occurring during a thyroid storm and chronic high-altitude hypoxia. The main suggestion is that chronic hypobaric hypoxia may act as an additional extra environmental stressor which, in combination with severe thyrotoxicosis increases sympathetic activation, enhances myocardial sensibility to catecholamine, impairs coronary microvascular function, and, ultimately, leads to Takotsubo syndrome and its thrombotic complications. Jiménez-Jiménez et al. discussed the onset of Takotsubo syndrome during a cardiac rehabilitation session after a recent non-ST-segment elevation myocardial infarction, highlighting structured physical exercise as an unusual but relevant trigger of stress-induced myocardial dysfunction. Tang et al. reported a case of diffuse multivessel coronary artery spasm presenting with cardiac arrest, a brief return of spontaneous circulation, and ST-segment elevation mimicking acute myocardial infarction. Angiographic confirmation showed severe but reversible multivessel stenoses that responded to intracoronary nitroglycerin. Finally, Milosevic et al. reported a rare case of Parkes-Weber syndrome in a 73-year-old patient with concomitant coronary artery disease and borderline left ventricular ejection fraction who underwent coronary artery bypass grafting. This case underlines the diagnostic and therapeutic challenges of managing combined congenital vascular malformations and acquired ischemic heart disease.

Chest pain is not restricted to myocardial ischemia and coronary vasomotor disorders. Inflammatory myocardial and pericardial diseases may present with infarct-like chest pain or electrocardiographic abnormalities, thereby closely mimicking an acute coronary syndrome even in the absence of obstructive coronary artery disease (6). Although cardiac magnetic resonance (CMR) increased the proportion of patients diagnosed with acute myocarditis from 5% to 13% among patients presenting with angina-like symptoms and elevated high-sensitivity cardiac troponin T, this condition still remains under-recognized, because of its heterogeneous clinical manifestations and the wide spectrum of potential triggers (7). Accordingly, myocarditis should also be considered in complex systemic presentations in which the underlying inflammatory or immune-mediated association may be atypical. In this context, Ayish et al. reported a rare case of eosinophilic granulomatosis with polyangiitis (EGPA) presenting with acute heart failure secondary to eosinophilic myocarditis. Immuno-suppressive treatment with high-dose corticosteroids and cyclophosphamide improved her condition and led to partial recovery of cardiac function. Similarly, Fu et al. described an unusual, rare case of myocarditis occurring concomitantly with acute pancreatitis and transient thyrotoxicosis, in which the concurrent involvement of the pancreas, thyroid, and myocardium might reflect a shared inflammatory or immune-mediated mechanism. In these cases, accurately identifying the pathophysiological mechanisms underlying myocarditis could help tailor the treatment.

Pericarditis represents another relevant inflammatory condition in the differential diagnosis of acute chest pain, accounting for ∼5% of emergency department evaluations for this symptom (6). Although most cases in first-income countries are classified as idiopathic or presumed to be of viral origin, specific infectious etiologies should not be overlooked, particularly in patients with immunosuppression, systemic manifestations, or an atypical clinical course (6, 8). Wang et al. described a 55-year-old woman with extrapulmonary tuberculosis presenting with a haemorrhagic pericardial effusion and multiple large caseous pericardial masses, an exceptionally rare manifestation that initially raised suspicion of a pericardial malignancy. Zhong et al. reported purulent pericarditis caused by Nocardia in a patient with acquired immunodeficiency syndrome (AIDS). The condition was initially misdiagnosed as tuberculous pericarditis, but the lack of clinical response prompted microbiological analysis of the pericardial fluid, which ultimately led to the identification of Nocardia and the initiation of targeted antimicrobial treatment. Together, these cases emphasize the need to consider uncommon and opportunistic pathogens in pericardial disease, particularly when the presentation, host characteristics, or response to empirical treatment is atypical.

Another major cardiovascular condition associated with substantial morbidity and an increased risk of sudden cardiac death, particularly in younger individuals, is hypertrophic cardiomyopathy (HCM). HCM is the most common inherited cardiomyopathy and is characterized by otherwise unexplained left ventricular hypertrophy, frequently accompanied by diastolic dysfunction, dynamic left ventricular outflow tract obstruction, myocardial ischemia, and potentially life-threatening arrhythmias. Its clinical presentation is highly heterogeneous, ranging from an asymptomatic phenotype to advanced heart failure or sudden cardiac death. Liu et al. described elderly HCM patients with sinus node dysfunction and pericardial effusion successfully managed with dual-chamber pacing. This intervention improved rhythm control and reduced left ventricular outflow tract obstruction, illustrating the potential value of individualized device-based strategies in selected patients with complex clinical presentations. From a precision medicine perspective, substantial progress has been made in elucidating the genetic basis of HCM, leading to the identification of more than 900 genetic variants across more than 20 genes, many of which involve gene encoding sarcomeric proteins, including myosin-7, encoded by myosin heavy chain (MYH) genes (9). Li et al. reported an interesting case report of a HCM patient with pathogenic MYH7 variant whose therapy was personalized by switching from diltiazem to metoprolol, alongside recommendations for low-intensity exercise, genetic screening of relatives, and pharmacist-supported long-term management. Hamidi et al. described an exceptional case where wild-type transthyretin amyloidosis coexisted with a rare MYH6/MYH7 duplication. This illustrates how overlapping genetic and infiltrative mechanisms can converge to produce an extreme and complex HCM phenotype. Similarly, Chumakova et al. reported three cases of HCM mimics, caused by variant in amyloidosis, Fabry disease, and desminopathy related genes, underlying the challenging of this diagnosis, particularly in those patients with age-related conditions that can mimic the clinical manifestations of HCM.

Beyond their clinical direct manifestation, ischemic, inflammatory, and congenital cardiac disorders may culminate in cardiac rupture or abnormal fistulous communications. These complications are uncommon but potentially catastrophic, as they may cause haemopericardium, cardiac tamponade, pathological shunting, or systemic embolization (10). Post-infarction ventricular free-wall rupture occurs in fewer than 1% of patients with acute myocardial infarction; nevertheless, mortality approaches 90% with conservative management, while operative mortality remains approximately 30% even among patients undergoing surgical repair. Recent case reports illustrate the markedly heterogeneous clinical settings in which cardiac wall disruption may occur. Chen et al. reported a 22-year-old woman with a congenital left ventricular diverticulum arising from the left ventricular outflow tract and rupturing into the pulmonary artery, thereby creating an abnormal communication. Liu et al. described the case of a 47-year-old middle-aged male with post-myocardial infarction free wall rupture with massive hemorrhagic pericardial effusion. Finally, Dai et al. reported a 71-year-old man with atrioesophageal fistula (AEF) after radiofrequency ablation for atrial fibrillation, complicated by ischemic stroke and myocardial infarction. Together, these cases illustrate how catastrophic disruption of cardiac wall integrity may result from congenital malformation, ischaemic necrosis, or iatrogenic injury.

Loss of structural integrity is not confined to the cardiac wall but may also involve the great vessels, particularly the aorta. Vasculopathies represent aheterogeneous group of disorders associated with structural and functional defects of blood vessels leading to impaired vascular homeostasis, inflammation, thrombosis, and tissue ischemia (11). They may involve vessels of different sizes and can result from genetic, autoimmune, inflammatory, infectious, or iatrogenic mechanisms (12). Chinaliyev et al. reported a rare case of ruptured abdominal aortic aneurysm (AAA) complicated by an infected retroperitoneal hematoma in a 59-year-old man who presented with persistent low back pain, fever, and impaired mobility. Diagnostic imaging revealed a ruptured infrarenal saccular AAA, which required emergency surgical intervention involving extra-anatomic bypass and aneurysm resection. Despite intraoperative complications, the patient achieved clinical recovery after intensive care and demonstrated promising outcomes during follow-up. Ronen et al. reported an ascending aortic pseudoaneurysm following 9 months from coronary artery bypass graft surgery in a 59-year-old man. In this case, because conventional surgery was considered to carry a high operative risk, the Heart Team recommended percutaneous endovascular repair, which resulted in significant hemodynamic improvement, suggesting that percutaneous endovascular treatment may be feasible in medical emergencies of the ascending aorta, when conventional surgery carries prohibitive risk.

In conclusion, the case reports included in this Research Topic broaden our understanding of several areas of cardiovascular medicine by highlighting unusual clinical presentations, diagnostic pitfalls, uncommon disease associations, and individualized therapeutic strategies. Collectively, they emphasize the continuing value of case reports in improving clinical awareness, supporting patient-centred decision-making, and generating hypotheses for further research.
